# Resveratrol prevents gallstones in mice fed on a high fat diet via regulating PPAR-γ and SR-BI

**DOI:** 10.3389/fphar.2025.1543865

**Published:** 2025-03-17

**Authors:** Menglu Zhao, Boya Xie, Yuxuan Li, Haiqing Dong, Sijia Jiang, Tiantian Zhu, Xiaolong Wu, Chengchen Xu, Jian Zhang, Shiyi Sun, Rui Li, Yinghai Xie

**Affiliations:** ^1^ School of Medicine, Anhui University of Science and Technology, Huainan, Anhui, China; ^2^ Anhui Shendong Biotechnology Development Co., Ltd., Huainan, China; ^3^ Department of Hepatobiliary Surgery, The First Affiliated Hospital of Anhui University of Science and Technology, Huainan, Anhui, China; ^4^ Department of Burn and Plastic Surgery of the First Affiliated Hospital of Nanjing Medical University, Nanjing, China

**Keywords:** resveratrol, cholesterol gallstones, PPARγ, bile cholesterol saturation, SR-BI, C57BL/6 mice

## Abstract

**Background:**

With the gradual improvement of living standards, the incidence of gallstones is getting higher and higher, and cholesterol gallstones (CG) are the most prevalent subtype. Therefore, we urgently need a better way to treat gallstones.

**Objective:**

This study aimed to evaluate the effects of resveratrol (Res) on cholesterol gallstone formation and explore its underlying mechanisms, focusing on its modulation of hepatic peroxisome proliferator-activated receptor γ (PPAR-γ) expression, bile cholesterol saturation, and hepatic cholesterol metabolism.

**Methods:**

Thirty-two male C57BL/6 mice were randomly divided into four groups: control, model, ursodeoxycholic acid (UDCA), and Res groups. Res (100 mg/kg/day) and UDCA (100 mg/kg/day) were administered via gavage for 5 weeks. Gallbladder bile, liver, and gallbladder tissues were collected for bile cholesterol crystal analysis, bile lipid profiling, and histopathological examination. Protein expression levels of PPARγ and scavenger receptor class B type I (SR-BI) were analyzed using Western blotting and immunohistochemistry.

**Results:**

Mice fed on a high fat diet resulted in larger gallbladder (about 2 times in both long and width diameters compared to control group) and CG formation, while resveratrol treatment significantly reduced gallstone formation, improved gallbladder dilatation, and declined cholestasis symptoms. Res suppressed hepatic inflammation by downregulating the receptor for advanced glycation end products (RAGE) expression and inhibiting the synthesis of proinflammatory factors. Res alleviated liver lipid deposition. It also enhanced PPARγ and SR-BI expression, promoting cholesterol efflux and lowering cholesterol levels, thereby preventing CG formation in mice.

**Conclusion:**

Resveratrol demonstrates significant potential as a therapeutic agent for the prevention and treatment of cholesterol gallstone disease (CGD) by modulating hepatic cholesterol metabolism, reducing bile cholesterol saturation, and alleviating hepatic inflammation. Further studies are warranted to explore its clinical applicability in humans.

## Introduction

Cholesterol gallstone disease is a widespread condition characterized by the formation of cholesterol-rich stones in the gallbladder, posing a significant global public health challenge (Wen et al., 2024). Affecting 10%–15% of adults in Western populations, its prevalence is higher among women, individuals with obesity, and those with diabetes or hyperlipidemia. In Asia, rising rates of obesity and metabolic syndrome have led to an increased prevalence, reflecting the influence of changing diets and lifestyles (Pan and Zhu, 2024). The disease imposes substantial economic and health burdens, with over one million hospitalizations annually in the United States alone, contributing billions in direct medical costs (Unalp-Arida and Ruhl, 2024; Stinton et al., 2010). Additionally, the condition causes indirect costs through lost productivity and reduced quality of life (Wang et al., 2024; Dehkharghani et al., 2003). Symptoms range from biliary colic to severe complications such as cholecystitis, pancreatitis, and cholangitis, which can be life-threatening (Ciorba et al., 2016). The need for invasive treatments and recurring symptoms often exacerbate anxiety and diminish patient wellbeing (Helder et al., 2001).

The pathophysiology of CGD is complex and multifactorial, involving genetic, metabolic, and environmental factors (Di Ciaula et al., 2018). Central to its development is bile supersaturation with cholesterol (Chen et al., 2015). Under normal conditions, bile—a mixture of bile acids, cholesterol, phospholipids, and bilirubin—maintains a delicate balance that prevents cholesterol precipitation (Méndez-Sánchez et al., 1996). However, excessive cholesterol secretion by the liver or impaired clearance disrupts this balance, leading to cholesterol crystal formation and eventual gallstone development. Genetic predispositions affecting bile acid synthesis, cholesterol metabolism, and gallbladder motility play significant roles, while environmental factors, including high-fat diets and obesity, exacerbate the condition (Di Ciaula et al., 2018; Fleishman and Kumar, 2024). Obesity, insulin resistance, and dyslipidemia increase cholesterol secretion and decrease its clearance from bile, promoting supersaturation (Méndez-Sánchez et al., 2007; Mc Auley, 2020). Additionally, diabetes and certain medications can impair gallbladder motility, causing bile stasis and further concentrating cholesterol, thereby increasing gallstone risk. The combined impact of these metabolic abnormalities significantly increases the risk of CG, so metabolic health issues need to be addressed to effectively prevent and manage CG.

Medical treatments for CG primarily focus on dissolving stones or preventing their formation (Bagepally et al., 2021). Ursodeoxycholic acid (UDCA), a widely used therapy, reduces cholesterol saturation in bile and increases bile fluidity, enabling the dissolution of small cholesterol stones. However, its effectiveness is limited, particularly for larger stones, and often requires prolonged treatment lasting months or even years (Madden et al., 2017). Additionally, UDCA does not address the underlying metabolic factors contributing to gallstone formation. Other medications, such as chenodeoxycholic acid and lithocholic acid, have shown variable success. Surgical intervention, typically cholecystectomy (gallbladder removal), remains the standard treatment for symptomatic gallstones but carries risks, including infection, bile duct injury, and the potential need for further surgeries (Li X. et al., 2024). These limitations highlight the urgent need for new therapeutic strategies that target the root causes of cholesterol gallstone disease rather than merely managing its symptoms.

In recent years, natural compounds such as resveratrol have garnered attention for their potential therapeutic effects on cholesterol gallstones by modulating cholesterol metabolism and bile composition (Vikal et al., 2024). Resveratrol, a polyphenolic compound found in grapes and various plants, is known for its antioxidant, anti-inflammatory, and lipid-lowering properties (Li Z. et al., 2024). It has been shown to regulate cholesterol metabolism through mechanisms such as the activation of AMP-activated protein kinase (AMPK), a key regulator of cellular energy balance. AMPK activation inhibits hepatic cholesterol synthesis and promotes fatty acid oxidation, potentially reducing gallstone formation. Additionally, resveratrol’s antioxidant effects may mitigate oxidative stress, a factor implicated in dyslipidemia and other metabolic disorders that contribute to gallstone development. Another promising pathway is the peroxisome proliferator-activated receptor (PPAR) pathway (Lan et al., 2017). PPARγ plays a key role not only in lipid metabolism but also in the regulation of hepatic inflammatory diseases, such as nonalcoholic fatty liver disease (NAFLD) (Han et al., 2019; Wen et al., 2022). Studies suggest that activating PPARγ can reduce the production of pro-inflammatory cytokines and suppress inflammatory pathways, including NF-κB, which are implicated in gallstone development. Additionally, receptors like RAGE (receptor for advanced glycation end-products) on hepatocytes activate inflammatory pathways that contribute to gallstone formation. Modulating PPAR signaling may help alleviate inflammation and improve bile composition, presenting a potential therapeutic approach for CGD.

At present, there are not many reports about whether resveratrol can be used to treat gallstones and its mechanism of action. Based on this, this study integrated and designed an experiment that aimed to explore whether PPAR-γ could reduce inflammation and prevent gallstones by regulating the expression of NF-kB pathway and RAGE, to achieve the purpose of alleviating CGD, which is expected to provide potential targets and experimental evidence for the treatment of traditional Chinese medicine for CGD.

## Materials and methods

### C57BL/6 mouse gallbladder stone model construction

All procedures followed the guidelines of the NIH Guide for the Care and Use of Laboratory Animals (NIH Publication 8023, revised 1978) and were approved by the Institutional Animal Care and Use Committee of the College of Medicine, Anhui University of Science and Technology (LW-2022-001, 2022.10.20) (Table 1).

**TABLE 1:** Study design.

Groups	Diet	Treatment	Mice number
Normal	basal feed	None	8
HF	high fat	None	8
UDCA + HF	high fat	ursodeoxycholic acid	8
Res + HF	high fat	Resveratrol	8

SPF 6-week-old male C57BL/6 mice (license number: SCXK (Su) 2021–0013) were purchased from Jiangsu Cavens Laboratory Animal Co., Ltd. 32 mice were randomly divided into 4 groups after 1 week of adaptation, namely normal group, HF group, UDCA + HF group and Res + HF group (n = 8 mice per group see Table 1). Mice in Normal group were fed with basal feed, while mice in high fat (HF) group were fed with high-fat, high-cholesterol lithogenic feed. UDCA + HF group and Res + HF group were treated groups. Mice in UDCA + HF group were fed a stone-inducing diet and UDCA (100 mg/kg/d) (Tang et al., 2016), and mice in Res + HF group were fed a stone-inducing diet and resveratrol (100 mg/kg/d) (Shen et al., 2023). Ursodeoxycholic acid was dissolved in saline, and resveratrol powder was dissolved in 0.5% sodium carboxymethylcellulose solution (Fracasso et al., 2021). Normal group and HF group were given equal volume of 0.5% sodium carboxymethylcellulose solution every day.

Specifically, the HF group received a diet consisting of high levels of cholesterol and fat (10% lipid, 1% cholesterol, and 0.5% cholate; TP 06116F1, Trophic Animal Feed High-tech Co. Ltd., Nantong, China) (Wang et al., 2022), and the control group was fed a standard diet (LAD0011). Ursodeoxycholic acid and resveratrol used in the treatment group were purchased from Chengdu Prifa Technology Development Co., LTD. Sodium carboxymethyl cellulose was purchased from Shanghai Maclin Biochemical Technology Co., LTD. All mice were housed in SPF mouse independent ventilation cages, with 4 mice in one cage and a total of 8 mouse cages. Animals were grown under standard laboratory conditions (12 h of light, 12 h of darkness, 21°C–24°C, and 50%–55% humidity), with 20 g of food per cage per day, changed daily, all with free access to water, and gavaged once daily. In order to alleviate the pain of the mouse, the needle should be inserted through the mouth of the mouse, allowing the needle to press down the mouse’s tongue and enter the esophagus as quickly as possible.

### Model assessment

After feeding for 5 weeks, 5 mice in the model group were randomly selected, the gallbladder was extracted, bile was collected, and the presence of sediment or granular deposits in the gallbladder and extrahepatic bile ducts of the mice could be recognized as gallstones by naked eye observation.

### General observation

The mice were weighed and recorded once a day in the evening. During the feeding period, the mice were observed daily for their mental state, appetite, fur color and other growth conditions, as well as for any adverse reactions.

### Specimen collection

5 weeks later, the mice were anesthetized by intraperitoneal injection of 4% chloral hydrate (300 mg/kg) and blood was collected from the eyeballs. The drawn blood was left at room temperature for 30 min and centrifuged at 4°C for 1500 r/min for 15 min to obtain the upper serum layer for subsequent biochemical assays. After photographing the overall appearance of the mice the liver, gallbladder and bile from the bile duct were collected from each mouse and sequentially photographed and observed, the long and wide diameters of the gallbladder were measured with vernier calipers and recorded, and the wall of the gallbladder was measured by microscopic observation. The livers were weighed. The liver near the gallbladder part, was taken and put into 4% paraformaldehyde tissue fixative and stored at room temperature; the rest of the livers were quick-frozen in liquid nitrogen and stored at −80°C. The collected gallbladder was put into 4% paraformaldehyde tissue fixative and stored at room temperature; the collected bile was put into PCR tubes and stored at −80°C; the volume of the gallbladder was calculated according to the elliptic formula: volume (ul) = length (mm)*width (mm)*depth (mm)*π/6, which is approximated by the fact that the width of the gallbladder is equal to the diameter of the depth of the gallbladder.

### Bile cholesterol crystal analysis

One drop of bile was taken from the gallbladder of 5 mice each from the control group, model group, ursodeoxycholic acid group and resveratrol group randomly, and a smear was made. The smear was made as follows. Place a drop of bile on one end of the slide, place a smooth-edged slide in front of the drop of bile, and slowly move it backward until it touches the bile. Make the bile liquid evenly dispersed to the contact between the slide and the slide, then keep the slide and the slide at an angle of 30°–40° and push the slide to the other end smoothly and evenly, leaving a thin film of bile on the slide. After the bile smear was pushed, it quickly shaken in the air and allowed to dry naturally to avoid deformation of the gallbladder cells. After the bile film was dried, a number was written on one side of the bile film with a pencil, and the cholesterol crystallization in mouse bile was observed under a polarized light microscope.

### Bile lipid analysis

Determination of cholesterol, total bile acids and phospholipids in bile fluid, total bile acids and phospholipids in bile fluid were measured by semi-automatic biochemical analyzer using enzymatic total cholesterol assay kit, total bile acid assay kit, and enzymatic phospholipid assay kit (Shanghai HUZHEN Industry Co., Ltd.), respectively, according to the protocols of the corresponding manufacturers (Silvain et al., 2012).

### Preparation of paraffin sections

The liver was rinsed with saline, fixed with 4% paraformaldehyde for 20 min in a water bathat 60°C, dehydrated in 75%, 95%, and absolute ethanol for 20min, 45min, and 45 min sequentially, then transparent in xylene for 20 min, infiltrated with paraffin in a paraffin bath (melting point: 52°C–54°C) for 20 min, and embedded in paraffin. After solidification, the paraffin block was sectioned into 5 μm thick slices and stored for future use.

The gallbladder was rinsed with saline, fixed with 4% paraformaldehyde for 5 min in a water bathat 60°C, dehydrated in 75%, 95%, and absolute ethanol for 5 min sequentially, then ransparent in xylene for 5 min, infiltrated with paraffin in a paraffin bath (melting point: 52°C–54°C) for 20 min, and embedded in paraffin. After solidification, the paraffin block was sectioned into 5 μm thick slices and stored for future use.

### Hematoxylin and eosin (H&E) staining

The paraffin-embedded liver sections were dewaxed twice in xylene solution for 30 min each time and hydrated in descending alcohol series for 5 min each and rinsed with water for 3 min. Then the paraffin sections were stained with hematoxylin for 10 min at room temperature, washed with water for 3 min, and differentiated with 0.1% hydrochloric acid in ethanol for 4 s, washed with water for 3 min, and returned to the blue solution for 4 s and washed with water for 3 min the sections were then stained with hematoxylin for 5 min in 85% ethanol, 95% ethanol, and washed with eosin at room temperature. The samples were stained with eosin for 1 min at room temperature, dehydrated in 100% ethanol for 5 min, and then cleared twice in xylene solution for 5 min each. The slices were finally sealed and finally mounted with neutral gum for microscopic observation (Lee et al., 2020).

The paraffin-embedded gallbladder sections were deparaffinized in xylene solution twice (first for 5 min, second for 10 min), followed by hydration in a descending alcohol series: 100% ethanol for 10 min, 95% ethanol for 3 min, and rinsed with water for 3 min. The sections were then stained with hematoxylin at room temperature for 10 min, rinsed with water for 15 s, differentiated with 0.1% hydrochloric acid ethanol for 8 s, rinsed again with water for 5 s, treated with bluing solution for 6 s, and rinsed once more with water for 5 s. Subsequently, the sections were counterstained with eosin at room temperature for 45 s, dehydrated in 95% ethanol for 10 min and 100% ethanol for 7 min, cleared in xylene solution twice (5 min each), and finally mounted with neutral gum for microscopic observation (Dai et al., 2023).

### Masson staining

The paraffin-embedded gallbladder sections were deparaffinized twice in xylene solution for 30 min each time, and hydrated in a descending sequence of alcohol series for another 5 min each, and then the paraffin sections were stained with Weigert’s ferric hematoxylin for 10 min (Weigert’s ferric hematoxylin is prepared now and used now: Weigert’s ferric hematoxylin A liquid and B liquid are mixed in equal quantities), washed in running water for 30 s, and differentiated with hydrochloric acid in alcohol for 15 s, 0.1% ammonia 15 s, washed with running water for 5 min, Liping Chunhong for 5 min (if Liping Chunhong is found to be too dark after dyeing, Liping Chunhong can be diluted by 2–4 times), washed with weak acid solution for 1 min (2 mL acetic acid plus 1000 mL of water), washed with molybdenum phosphate solution for 2 min, washed with weak acid solution for 1 min, solid green staining solution (aniline blue) for 2 min, washed with weak acid solution again for 1 min. Finally, the sections were dehydrated in 95% ethanol for 12 s and 100% ethanol twice (12 s each), cleared, and mounted with neutral gum for microscopic observation (Gautier et al., 2007).

### Immunohistochemistry (IHC) staining

The liver sections with a thickness of 5 microns were dewaxed twice in xylene for 30 min each time, and then hydrated in 100%, 95%, 85% and 75% alcohol for 5 min each, and then washed in ddH2O for 5 min: Incubate at room temperature with a membrane breaking solution (omit this step if the target antigen is on the cell surface) for 30 min. Then it was cleaned with PBST for 3 times, 5 min each time, and then treated with 3% hydrogen peroxide at room temperature and away from light for 30 min. After cleaning, rinse with ultra-pure water, repair with antigen repair solution for 30 min, and wash with ultra-pure water and PBST twice, 5 min each time after cooling. Then it was closed with 5% BSA for 2 h, and appropriate amount of anti-PPAR was added as needed (1:200,000,60127-1-AP; proteintech, USA), IL-6 (1:200, GB11117; Servicebio, China), RAGE (1:100, bs-0177R; Bioss, China) primary antibody and incubated at 4°C overnight. The next day, after rewarming at room temperature for 45 min, the primary antibody was recovered and washed 4 times with PBST solution for 5 min each time. Goat anti-rabbit coupled with horseradish peroxide (1:200, GB23303; Servicebio, China) or Goat anti-mouse secondary antibody (1:200, GB23301; Servicebio, China), incubated at room temperature for 1.5 h, then washed with PBST twice for 5 min each time. Add DAB color developing solution (G1212-200T, Servicebio China) for 8 min of color development, monitor the color reaction under the microscope, and terminate the water flushing in time. Re-dye with hematoxylin solution for 30 s, then rinse off. Reverse blue in PBST for 5 min. Then soak in 75%, 85%, 95%, 100% ethanol for 5 min, and xylene for 10 min respectively. Finally, the seal was fixed with neutral gum and observed under microscope to evaluate the localization and expression intensity of target protein (Yang et al., 2024). Note: All antibodies were diluted with PBST. During the histological analysis, 6 images were obtained from each tissue section, and each section was viewed at the full field of vision, counted, and averaged. Pathological sections of 8 mice in each group were stained.

### Western blot analysis

Fresh liver tissue was removed from −80°C and total protein was extracted from liver tissue using RIPA (radioimmunoprecipitation assay) cleavage buffer (P0013B, Beyotime, Shanghai, China) containing 4% protease inhibitor according to instructions (1 mL per 100 mg of tissue): Centrifuge beads were added and grinded with a grinder. The ice was cracked for 60 min (vortex mixing for 10 s every 15 min), followed by centrifuge at 12,000 RPM at 4°C for 15 min to remove the supernatant. BCA (dicinchoninic acid) protein concentration detection kit (P0010S, Beyotime) was used to determine the protein concentration of the samples. The samples were heated at 95°C for 5 min after adding the sample buffer and then subjected to 10% SDS-PAGE (Sodium dodecyl sulphate-polyacrylamide gel) electrophoresis (80 V 30 min, 120 V 60 min). The protein was then transferred to PVDF (polyvinylidene fluoride) membrane (FFP24, Beyotime) for 60 min by electrotransfer solution at 400 mA. The membrane was closed at room temperature with TBST (Tris Buffered Saline with Tween ^®^ 20) containing 5% skim milk (GC310001,Servicebio, China) for 2 h, then combined with a primary antibody against PPAR (1:300,60127-1-AP; proteintech, United States), RAGE (1:1200, bs-0177R; Bioss, China), NF-kB (1:750,GB11997; Servicebio, China), IL-6 (1:750, GB11117; Servicebio, China), SR-BI (1:750,GB112562; Servicebio, China) and GAPDH (1:1500, GB11002; Servicebio, China) overnight at 4°C. On the next day, TBST was used to wash the film 3 times for 10 min each time, and the film was coupled with horseradish peroxide in goats against rabbits (1:3000, GB23303; Servicebio, China) or Goat anti-mouse secondary antibody (1:400, GB23301; Servicebio, China) incubated at room temperature for 2 h, and then washed the film with TBST three times for 10 min each time (the incubation of primary and secondary antibodies was oscillated in a shaker at low speed, and the washing was oscillated in a shaker at medium speed). The film was developed using an ECL (Enhanced Chemiluminescence) chemiluminescence substrate kit (G2014,Servicebio, China), and the bands were visualized using an ECL luminescence detection system (ChemiDoc XRS+, Bio-Rad). The gray value of the strip is calculated using ImageJ software and normalized to GAPDH. The relative expression level was 3 times (Xia et al., 2012).

### Statistical analyses

Data are the mean ± standard deviation (SD). Appropriate statistical analyses were applied depending on data distribution. For data that showed a normal distribution, the two-tailed Student’s t-test was used between two groups, and one-way analysis of variance followed by Tukey’s test for multiple comparisons was used between three or four groups. For datasets with a skewed distribution, the Mann–Whitney test was used between two groups, and the Kruskal–Wallis test followed by Dunn’s test for multiple comparisons was done between three or four groups. Statistical analyses were undertaken using Prism 9.5.

## Results

### Resveratrol reduces gallstones formation

**FIGURE 1:**
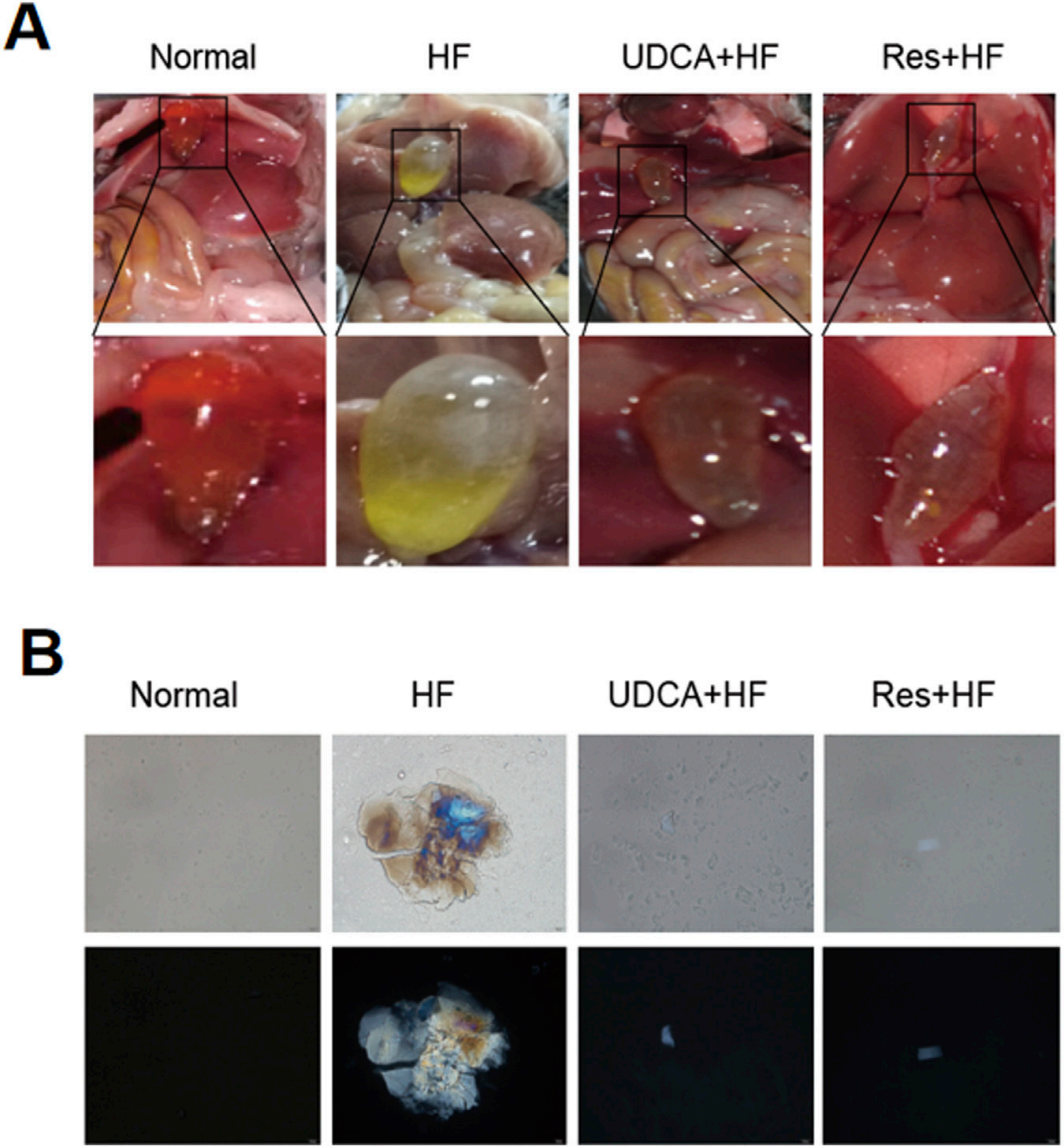
Resveratrol alleviates the occurrence of gallstones. **(A)** Representative images of gallbladder appearance from mice in the Normal, HF, UDCA + HF, and Res + HF groups. HF: high-fat diet, UDCA: ursodeoxycholic acid, Res: resveratrol. **(B)** Polarized light microscopic images showing bile crystals in the gallbladders of mice from each treatment group.

In this study, gallbladder stones in mice across all groups were observed as yellowish, granular, sandy, or irregularly shaped deposits, with bile clearly visible through the gallbladder wall. In the HF (high-fat diet) group, the gallbladders contained numerous large stones, and the bile appeared significantly more turbid compared to the other groups (Figure 1A). Conversely, the gallbladders of mice in the Normal, UDCA + HF (treated with ursodeoxycholic acid and HF, used as positive control), and Res + HF (treated with resveratrol and HF) groups contained only a few small stones, with bile that remained clear (Figure 1A).

Polarized light microscopy was used to analyze bile fluid crystals from each group (Figure 1B). The results revealed that bile fluid from the HF group contained a high number of typical cholesterol hydrate crystals, whereas the resveratrol and ursodeoxycholic acid groups exhibited only scattered cholesterol crystals. Moreover, the size and number of crystals in the UDCA + HF and Res + HF groups were significantly reduced compared to those in the HF group. These findings suggest that resveratrol effectively reverses gallstone formation and declines symptoms of gallbladder dilation and cholestasis to a degree comparable to ursodeoxycholic acid.

### Resveratrol alleviates gallbladder damage

**FIGURE 2:**
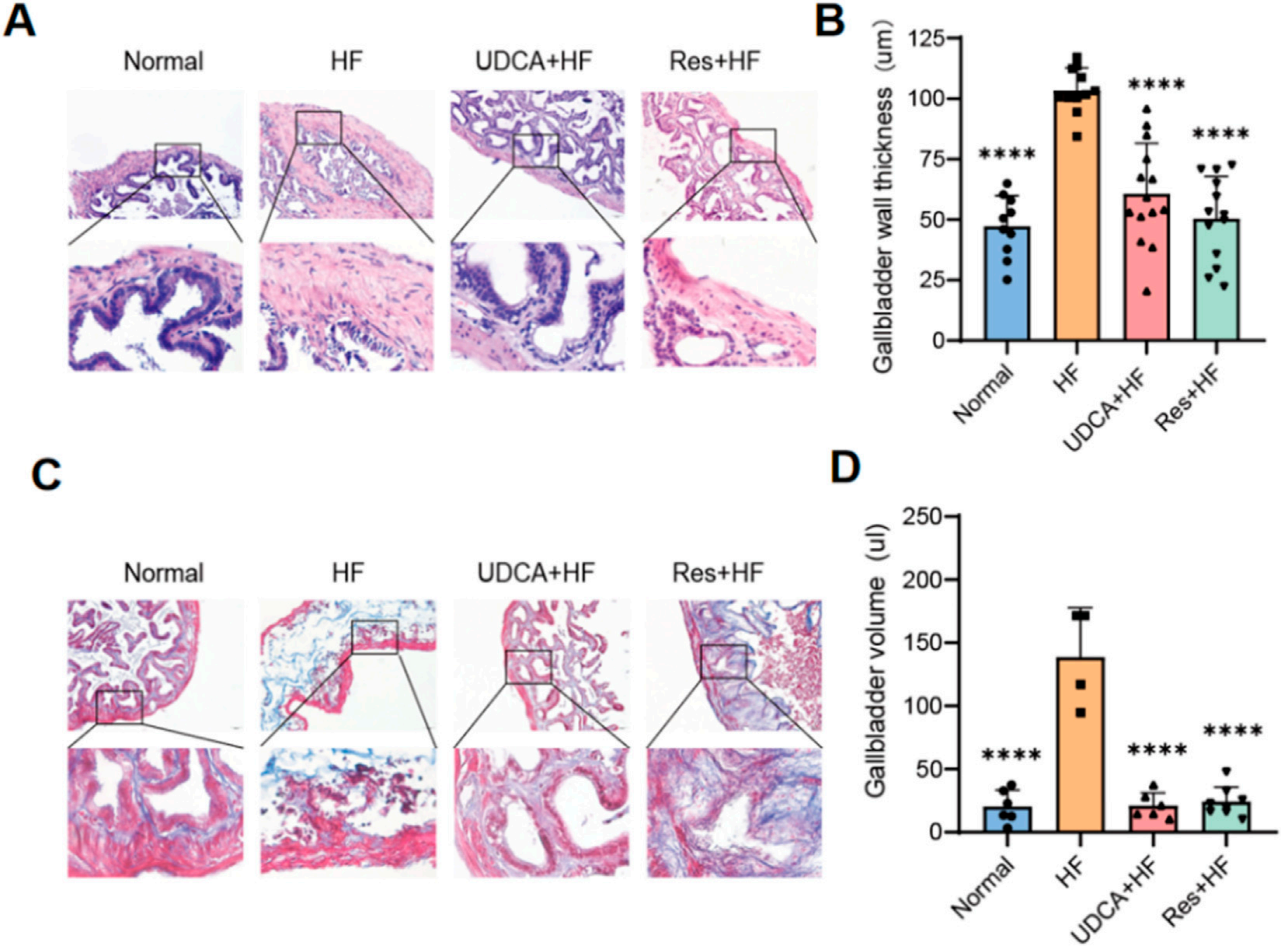
Resveratrol alleviates gallbladder injury in mice. **(A)** Representative histological images of gallbladder sections stained with hematoxylin and eosin (H&E) staining. The lower pictures are a magnification of the square area among the upper pictures. **(B)** Quantification of the gallbladder wall thickness in different experimental groups (n = 8). **(C)** Representative Masson’s trichrome staining showing collagen deposition in the gallbladder wall. The lower pictures are a magnification of the square area among the upper pictures. **(D)** Measurement of the gallbladder volume in different experimental groups (n = 6). *P < 0.05, **P < 0.01, ***P < 0.001, ****P < 0.0001.

Histological analysis of mouse gallbladder tissues using H&E staining revealed that the gallbladder tissues in the Normal group showed no signs of congestion or significant inflammatory cell infiltration (Figure 2A). In contrast, the HF group displayed extensive infiltration of inflammatory cells in the gallbladder tissue. Notably, the groups treated with ursodeoxycholic acid and resveratrol showed significant improvement, exhibiting minimal edema and only a small amount of inflammatory cell infiltration.

Furthermore, gallbladders from mice fed a high-cholesterol diet exhibited mucosal and connective tissue hyperplasia in the propria layer, along with a marked increase in small blood vessels and collagen fibers (Figure 2C). The HF group also displayed thicker gallbladder walls and larger gallbladder volumes (Figures 2B, D; Table 2). Importantly, these pathological changes were alleviated by treatment with ursodeoxycholic acid and resveratrol, which restored gallbladder wall thickness and structural integrity to levels comparable to the control group (Figure 2; Table 2).

**TABLE 2:** The gallbladder volume data.

Group	Gallbladder size (M±SD)		
	Long diameter (mm)	Width diameter (mm)	Volume (ul)
Normal	6.90 ± 0.74	2.50 ± 0.50	23.78 ± 11.20
HF	10.00 ± 1.15	5.13 ± 0.48	139.43 ± 39.82
UDCA + HF	6.58 ± 1.02	2.42 ± 0.49	21.03 ± 10.16
Res + HF	6.38 ± 1.16	2.63 ± 0.52	23.99 ± 11.75

### Resveratrol mitigates hepatic inflammation by suppressing RAGE production

**FIGURE 3:**
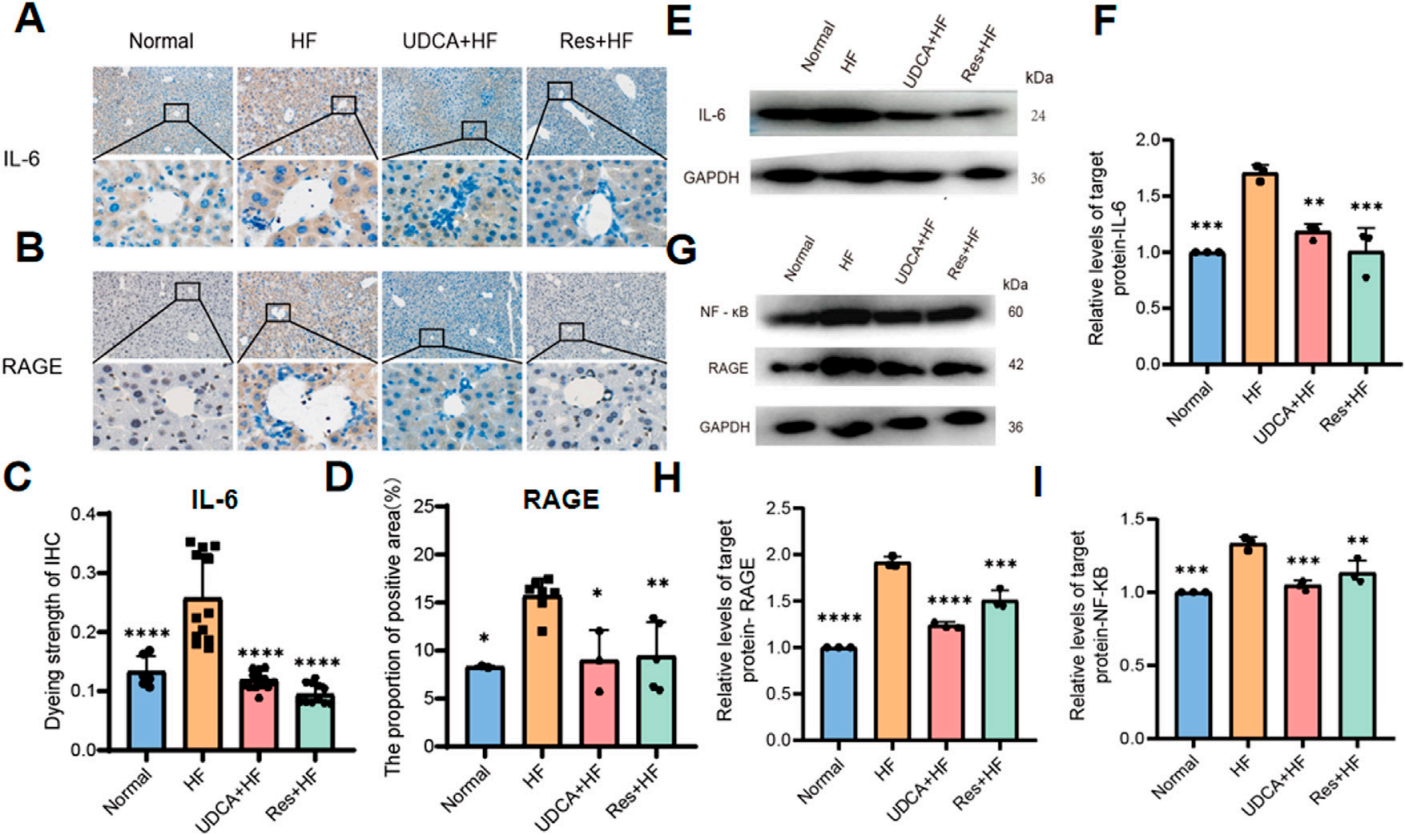
Resveratrol suppresses hepatic inflammation by inhibiting RAGE production. **(A)** Representative IHC staining IL-6 in liver tissues from four experimental groups. **(B)** Representative IHC staining for RAGE in liver tissues from four experimental groups. **(C)** Quantitative analysis of IL-6 staining intensity, presented as a histogram showing the enhancement in staining (n = 8). **(D)** Histogram depicting the percentage of the immunohistochemistry positive area of RAGE in liver tissues (n = 4∼6). **(E)** Western blot analysis of IL-6 protein levels in liver tissue samples. **(F)** Quantification of IL-6 protein levels expressed relative to control (n = 3). **(G)** Western blot analysis of NF-κB and RAGE protein expression in liver tissues. **(H)** Quantification of RAGE protein expression, relative to control (n = 3). **(I)** Quantification of NF-κB protein expression, relative to control (n = 3). HF: high-fat diet, UDCA: ursodeoxycholic acid, Res: resveratrol. Data are presented as mean ± SEM. Statistical significance was assessed by one-way ANOVA with Tukey’s *post hoc* test. *P < 0.05, **P < 0.01, ***P < 0.001, ****P < 0.0001.

Liver inflammation plays a pivotal role in the pathogenesis of gallstone formation. The receptor for advanced glycation end products (RAGE), upon binding to its ligands, activates various cellular signaling pathways, leading to the upregulation of the transcription factor NF-κB. This activation results in increased production of inflammatory cytokines and triggers an inflammatory response. Immunohistochemical analysis was performed to evaluate the expression levels of RAGE, the inflammatory cytokine IL-6, and NF-κB in mouse gallbladder tissues across different groups (Figures 3A–D). The results demonstrated a significant reduction in the expression levels of RAGE, IL-6, and NF-κB in the Res + HF and UDAC + HF groups compared to the HF group. Western blot (WB) analysis confirmed this trend (Figures 3E–I). These findings suggest that resveratrol may mitigate gallstone formation by downregulating RAGE expression and subsequently suppressing the synthesis of pro-inflammatory cytokines.

### Effect of resveratrol on lipid deposition and cholesterol saturation in the liver

**FIGURE 4:**
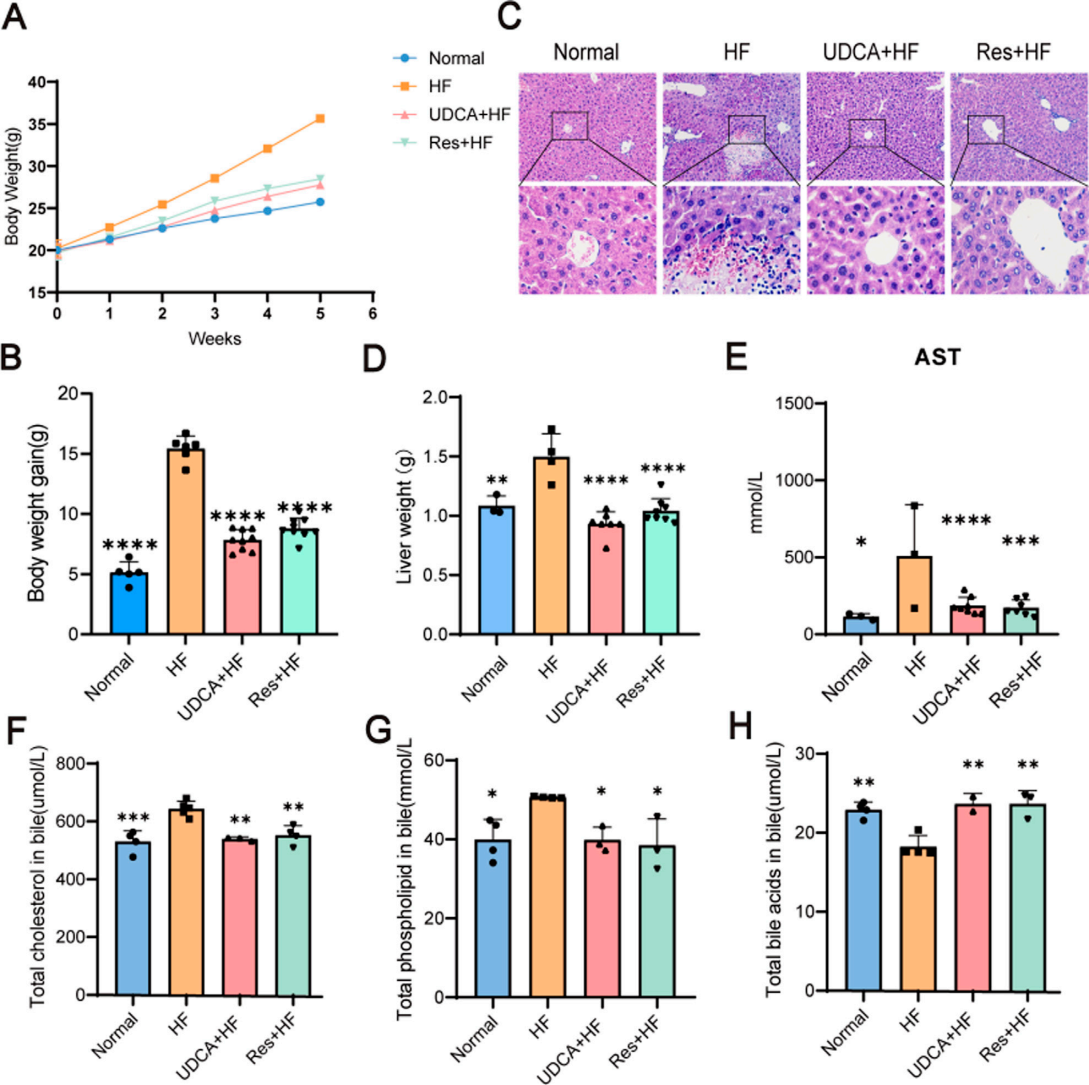
The therapy effects of resveratrol. **(A)** The body weight curves of mice in the Normal, HF, UDCA + HF, and Res + HF groups. **(B)** Total body weight gain in the different treatment groups over the study period (n = 5∼8). **(C)** Representative H&E staining images of liver tissues from mice in the four experimental groups. **(D)** Liver weight measurements in each treatment group (n = 5∼8). **(E)** Serum AST levels in the different groups (n = 5∼8). **(F)** Cholesterol content in the biliary system of mice (n = 4). **(G)** Phospholipid levels in the bile of treated and control mice. **(H)** Bile acid content in the bile fluid of mice. HF: high-fat diet, UDCA: ursodeoxycholic acid, Res: resveratrol. Data are presented as mean ± SEM. Statistical significance was assessed by one-way ANOVA with Tukey’s *post hoc* test. *P < 0.05, **P < 0.01, ***P < 0.001, ****P < 0.0001.

Body weight data collected over a 5-week period showed that mice in the HF group experienced significant weight gain compared to those in the Normal group (Figure 4A). A high-fat diet induced substantial body weight gain, whereas resveratrol effectively inhibited this increase (Figure 4B). Microscopic examination of liver tissue stained with H&E revealed that hepatocytes in the HF group were notably enlarged and exhibited pronounced fatty degeneration, accompanied by varying degrees of inflammatory cell infiltration (Figure 4C). Additional pathological features included hepatocyte enlargement, focal necrosis, fragmented necrosis, and bile stasis. In contrast, liver tissue lesions in mice treated with ursodeoxycholic acid or resveratrol were significantly milder compared to those in a high-fat environment. Mice fed a high-cholesterol diet, which promoted gallstone formation, displayed liver damage, including hepatomegaly. Notably, resveratrol treatment effectively mitigated the severity of liver damage.

As shown in Figure 4D, both UDCA and resveratrol significantly reduced liver weight, bringing it closer to or even slightly below the normal levels observed in the control group. Notably, the resveratrol group demonstrated a marked reduction in the liver weight-to-body weight ratio, indicating that the decrease in liver weight was independent of changes in body weight. This finding eliminates potential confounding effects of body weight on the data and highlights the specific impact of resveratrol on liver weight reduction.

Aspartate aminotransferase (AST) serves as a key biomarker for liver injury. Serum analysis from the mice revealed significantly elevated AST levels in the HF group compared to the control and treatment groups, providing further confirmation of liver damage (Figure 4E).

Bile cholesterol saturation is closely associated with cholesterol metabolism, and abnormalities in this process can alter bile cholesterol concentrations, leading to increased bile cholesterol saturation and a higher risk of cholesterol gallstone formation. In the treatment groups, cholesterol levels in the gallbladder bile of mice were significantly reduced (Figure 4F). Additionally, both the UDCA + HF and Res + HF groups demonstrated a reduction in bile phospholipid levels, bringing them closer to those observed in the control group (Figure 4G). Moreover, bile acid levels were significantly elevated in the UDCA + HF and Res + HF groups compared to the HF group, indicating that resveratrol promotes an increase in bile acid content in the bile of mice (Figure 4H).

### Resveratrol regulates PPAR-γ and SR-BI expression to improve cholesterol metabolism and prevent gallstones

**FIGURE 5:**
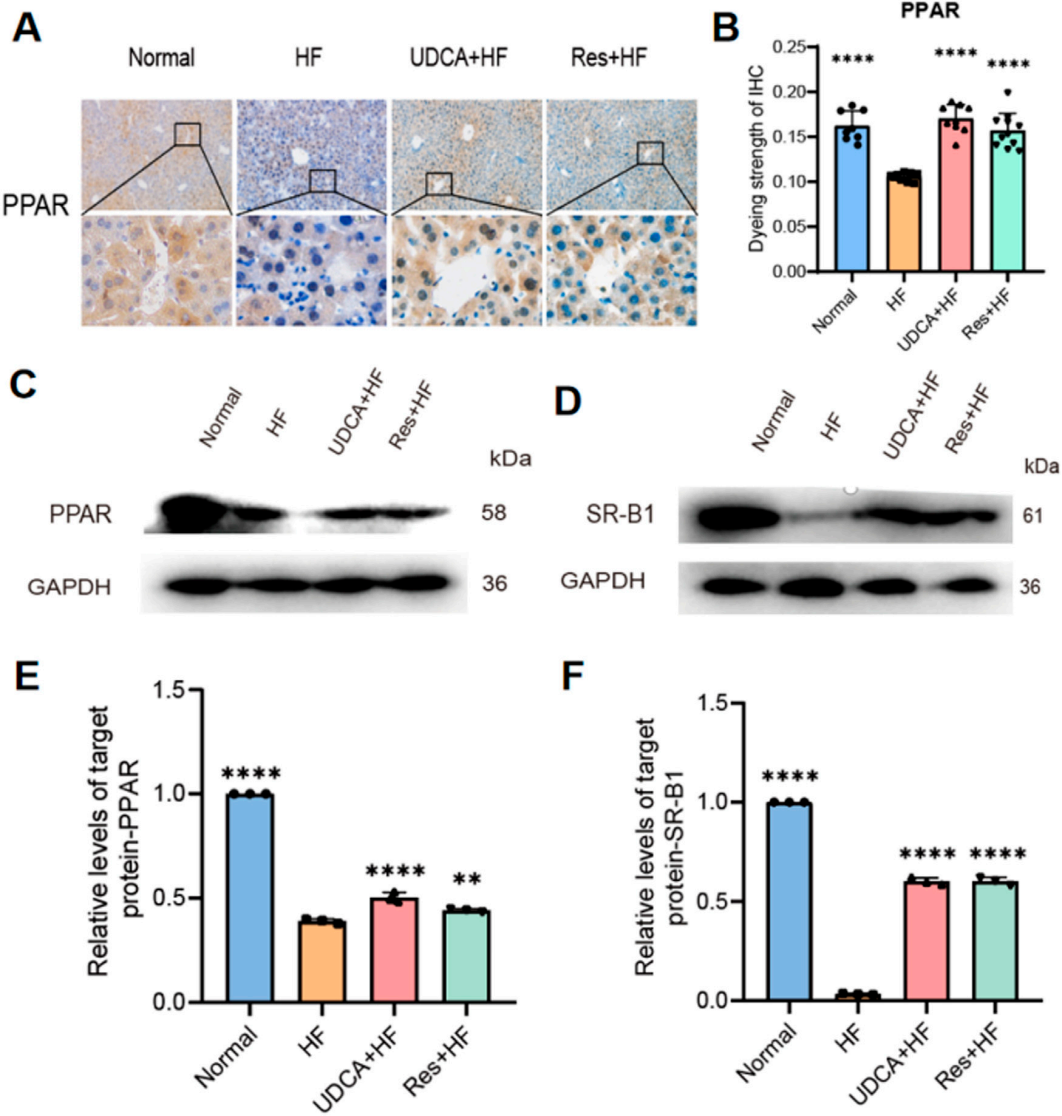
Effects of resveratrol on protein expression in hepatic cholesterol metabolism. **(A)** Representative IHC staining for PPAR-γ in liver tissues from the four experimental groups. **(B)** Quantification of PPAR-γstaining intensity, presented as a histogram (n = 8). **(C, D)** Western blot analysis showing the expression of **(C)** PPAR and **(D)** SR-BI protein. **(E)** Relative expression levels of PPAR protein from western blot analysis (n = 3). **(F)** Relative expression levels of SR-BI protein from western blot analysis (n = 3). HF: high-fat diet, UDCA: ursodeoxycholic acid, Res: resveratrol. Data are presented as mean ± SEM. Statistical significance was assessed by one-way ANOVA with Tukey’s *post hoc* test. *P < 0.05, **P < 0.01, ***P < 0.001, ****P < 0.0001.

Peroxisome proliferator-activated receptor (PPAR)-γ is a nuclear hormone receptor involved in regulating glucose homeostasis, lipid metabolism, and adipocyte function. Beyond its strong adipogenesis-promoting effects, PPAR-γ plays a pivotal role in reducing inflammation, oxidative stress, endoplasmic reticulum stress, and fibrosis. Previous studies have demonstrated that activating PPAR-γ can lower the risk of gallstone formation. In this study, PPAR-γ expression levels were assessed using Western blotting and immunohistochemistry. The results revealed a significant upregulation of PPAR-γ expression in the resveratrol-treated group, whereas its expression was notably decreased in the stone group (Figures 5A–C, E).

Scavenger receptor class B type I (SR-BI) plays a crucial role in cholesterol efflux. Numerous studies have shown that PPAR-γ can regulate SR-BI expression, thereby maintaining lipid and cholesterol homeostasis in hepatocytes and macrophages. Western blot analysis revealed a significant upregulation of SR-BI expression in the resveratrol-treated group, which enhanced cholesterol efflux, reduced cholesterol levels, and alleviated gallstone symptoms (Figures 5D, F).

## Discussion

Gallstones are not only a disorder of cholesterol metabolism but also a chronic inflammatory disease. Bile stasis can lead to inflammation in the liver and gallbladder. While inflammation is a protective response to injury, excessive inflammation can cause tissue and cell damage and death. The protective role of PPAR-γ in modulating inflammation has been well-documented in numerous studies. The overactivation of the PPAR-γ/NF-κB signaling pathway plays a significant role in the onset and progression of various inflammatory diseases, such as ARDS (acute respiratory distress syndrome), asthma, COPD (chronic obstructive pulmonary disease), and sepsis (Rozema et al., 2012). PPAR-γ is considered as a potent anti-inflammatory regulator. It modulates inflammatory signaling by interacting with transcription factors involved in the regulation of inflammatory cytokines, such as NF-κB, STAT, and AP-1, and its activation reduces the expression of pro-inflammatory cytokines. In the liver, Kupffer cells and recruited macrophages have been identified as key regulators of liver inflammation. Upon activation, Kupffer cells release inflammatory cytokines like IL-6, IL-1β, and TNF-α, which promote an inflammatory cascade. Chronic inflammation, in turn, contributes to liver fibrosis, characterized by the accumulation of extracellular matrix. Transforming growth factor-β (TGF-β) is a critical regulator of fibrosis, strongly stimulating hepatic stellate cells (HSCs) via Smad signaling. PPAR-γ directly binds to Smad3, inhibiting the TGF-β-induced expression of connective tissue growth factor (CTGF) and α-SMA (a marker of HSC activation), thereby suppressing HSC activation (Kökény et al., 2021). Our findings indicate that resveratrol pharmacologically activates PPAR-γ, downregulates the expression of inflammatory cytokine proteins such as NF-κB and IL-6, and alleviates liver and gallbladder inflammation. Furthermore, histological analysis using H&E staining reveals thinning of the gallbladder wall and a reduction in gallbladder inflammation. This dual function of SR-BI makes it crucial in regulating systemic cholesterol metabolism (Changizi et al., 2023).

PPAR-γ and LXRα are two key nuclear receptors crucial for lipid homeostasis and metabolic regulation. PPAR-γ, a member of the peroxisome proliferator-activated receptor family, plays a pivotal role in adipocyte differentiation and glucose metabolism. In contrast, LXRα is primarily involved in cholesterol efflux and the regulation of genes associated with lipid metabolism (Zhu et al., 2012; Yue et al., 2015; Hoekstra et al., 2010).The interplay between these nuclear receptors and SR-BI is vital for maintaining cholesterol balance within the body. For instance, SR-BI facilitates the selective uptake of HDL cholesterol in the liver, a crucial process in reverse cholesterol transport that helps prevent atherosclerosis (Hoekstra et al., 2010). Moreover, activation of LXRα has been shown to enhance the expression of ABCA1, a key transporter involved in cholesterol efflux, thereby linking LXRα signaling to the functional activity of SR-BI (Yue et al., 2015). Furthermore, the regulation of these receptors is influenced by various physiological and pathological conditions, including inflammation and metabolic disorders (Kamtchueng Simo et al., 2011). Understanding the mechanisms by which PPAR-γ and LXRα interact with SR-BI could provide valuable insights into potential therapeutic strategies for managing cholesterol-related diseases (Viennois et al., 2011; Liu et al., 2012). PPAR-γ upregulates the expression of LXRα either by directly binding to the PPRE in the LXRα promoter region or through other indirect pathways (Wang and Tall, 2003; Daffu et al., 2012).

Previous studies have shown that the PPAR-γ/LXRα pathway not only regulates ABCA1 but also plays a key role in controlling SR-BI expression. PPAR-γ enhances reverse cholesterol transport (RCT) by upregulating SR-BI. Additionally, research has indicated that RAGE ligands inhibit the activation of the PPAR-γ response element, thereby suppressing the promoter activity and transcription of ABCG1 and ABCA1, ultimately impairing cholesterol efflux. In this study, resveratrol was found to upregulate PPAR-γ, promote SR-BI expression, and downregulate RAGE, leading to enhanced cholesterol efflux, reduced cholesterol levels, and alleviation of gallstone formation.

Our study has several limitations. First, it did not perform an in-depth microscopic analysis of the gallbladder and did not list all abnormal findings, such as cells, crystals or other components. Second, although animal models can be used to study the process of stone formation, the pathogenesis of CG formation in mice and humans is not the same. For example, humans do not consume cholic acid through diet. These differences should be considered when translating research findings from mice to humans. In addition, we identified several potential targets of resveratrol for gallstone prevention, but further confirmation is needed.

## Conclusion

Our findings demonstrate that resveratrol significantly reduces gallstone formation and alleviates related symptoms, such as gallbladder dilatation and cholestasis. These effects are primarily mediated by the upregulation of hepatic PPAR-γ expression. Resveratrol-treated mice showed decreased bile cholesterol saturation, reduced bile phospholipid levels, and increased bile acid levels, indicating enhanced cholesterol excretion through improved bile acid synthesis or flow. Additionally, resveratrol suppressed hepatic inflammation by downregulating RAGE and inflammatory markers like IL-6 and NF-κB. It also mitigated high-fat diet-induced hepatic lipid deposition, as evidenced by reduced liver weights and improved histopathology. Importantly, these benefits were independent of body weight changes, with liver weight reductions aligned with control group levels.

In summary, these findings highlight the multifaceted mechanisms through which resveratrol may prevent cholesterol gallstone formation. By modulating hepatic cholesterol metabolism, lowering bile cholesterol saturation, and reducing hepatic inflammation, resveratrol shows great potential as a therapeutic candidate for the prevention and treatment of cholesterol gallstone disease. Further research is warranted to explore its clinical applicability in human patients with this condition.

## Data Availability

The original contributions presented in the study are included in the article/supplementary material, further inquiries can be directed to the corresponding authors.

## References

[B1] Bagepally B. S. Haridoss M. Sasidharan A. Jagadeesh K. V. Oswal N. K. (2021). Systematic review and meta-analysis of gallstone disease treatment outcomes in early cholecystectomy versus conservative management/delayed cholecystectomy. BMJ Open Gastroenterol. 8, e000675. 10.1136/bmjgast-2021-000675 PMC8280848 34261757

[B2] Changizi Z. Kajbaf F. Moslehi A. (2023). An overview of the role of peroxisome proliferator-activated receptors in liver diseases. J. Clin. Transl. Hepatol. 11, 1542–1552. 10.14218/JCTH.2023.00334 38161499 PMC10752810

[B3] Chen Y. Kong J. Wu S. (2015). Cholesterol gallstone disease: focusing on the role of gallbladder. Lab. Invest 95, 124–131. 10.1038/labinvest.2014.140 25502177

[B4] Ciorba A. Bianchini C. Scanelli G. Pala M. Zurlo A. Aimoni C. (2016). The impact of dizziness on quality-of-life in the elderly. Eur. Archives Oto-Rhino-Laryngology 274, 1245–1250. 10.1007/s00405-016-4222-z 27450383

[B5] Daffu G. Shen X. Senatus L. Thiagarajan D. Abedini A. Hurtado Del Pozo C. (2012). RAGE suppresses ABCG1-mediated macrophage cholesterol efflux in diabetes. Arterioscler. Thromb. Vasc. Biol. 23, 1178. 10.1161/01.ATV.0000075912 PMC4657581 26253613

[B6] Dai Y. Jia Z. Fang C. Zhu M. Yan X. Zhang Y. (2023). Polygoni Multiflori Radix interferes with bile acid metabolism homeostasis by inhibiting Fxr transcription, leading to cholestasis. Front. Pharmacol. 14, 1099935. 10.3389/fphar.2023.1099935 36950015 PMC10025474

[B7] Dehkharghani S. Bible J. Chen J. G. Feldman S. R. Fleischer A. B. Jr. (2003). The economic burden of skin disease in the United States. J. Am. Acad. Dermatology 48, 592–599. 10.1067/mjd.2003.178 12664024

[B8] Di Ciaula A. Wang D.Q.-H. Portincasa P. (2018). An update on the pathogenesis of cholesterol gallstone disease. Curr. Opin. gastroenterology 34, 71–80. 10.1097/MOG.0000000000000423 PMC8118137 29283909

[B9] Fleishman J. S. Kumar S. (2024). Bile acid metabolism and signaling in health and disease: molecular mechanisms and therapeutic targets. Signal Transduct. Target. Ther. 9, 97. 10.1038/s41392-024-01811-6 38664391 PMC11045871

[B10] Fracasso M. da Silva A. D. Bottari N. B. Monteiro S. G. Garzon L. R. de Souza L. A. F. (2021). Resveratrol impacts in oxidative stress in liver during Trypanosoma cruzi infection. Microb. Pathog. 153, 104800. 10.1016/j.micpath.2021.104800 33609651

[B11] Gautier T. Tietge U. J. Boverhof R. Perton F. G. Le Guern N. Masson D. (2007). Hepatic lipid accumulation in apolipoprotein C-I-deficient mice is potentiated by cholesteryl ester transfer protein. J. lipid Res. 48, 30–40. 10.1194/jlr.M600205-JLR200 17053273

[B12] Han T. Lv Y. Wang S. Hu T. Hong H. Fu Z. (2019). PPARγ overexpression regulates cholesterol metabolism in human L02 hepatocytes. J. Pharmacol. Sci. 139, 1–8. 10.1016/j.jphs.2018.09.013 30554802

[B13] Helder D. I. Kaptein A. A. van Kempen G. M. van Houwelingen J. C. Roos R. A. (2001). Impact of Huntington's disease on quality of life. Mov. Disord. 16, 325–330. 10.1002/mds.1056 11295789

[B14] Hoekstra M. Van Berkel T. J. Van Eck M. (2010). Scavenger receptor BI: a multi-purpose player in cholesterol and steroid metabolism. World J. Gastroenterol. 16, 5916–5924. 10.3748/wjg.v16.i47.5916 21157967 PMC3007109

[B15] Kamtchueng Simo O. Ikhlef S. Berrougui H. Khalil A. (2011). Advanced glycation end products affect cholesterol homeostasis by impairing ABCA1 expression on macrophages. World J. Gastroenterol. 17, 59–24. 10.3748/wjg.v16.i47 28704619

[B16] Kökény G. Calvier L. Hansmann G. (2021). PPARγ and tgfβ—major regulators of metabolism, inflammation, and fibrosis in the lungs and kidneys. Int. J. Mol. Sci. 22, 10431. 10.3390/ijms221910431 34638771 PMC8508998

[B17] Lan F. Weikel K. A. Cacicedo J. M. Ido Y. (2017). Resveratrol-induced AMP-activated protein kinase activation is cell-type dependent: lessons from basic research for clinical application. Nutrients 9, 751. 10.3390/nu9070751 28708087 PMC5537865

[B18] Lee E. S. Kwon M.-H. Kim H. M. Woo H. B. Ahn C. M. Chung C. H. (2020). Curcumin analog CUR5–8 ameliorates nonalcoholic fatty liver disease in mice with high-fat diet-induced obesity. Metabolism 103, 154015. 10.1016/j.metabol.2019.154015 31758951

[B19] Li X. Ouyang J. Dai J. (2024a). Current gallstone treatment methods, state of the art. Diseases 12, 197. 10.3390/diseases12090197 39329866 PMC11431374

[B20] Li Z. Liu S. Liu Q. Wang M. Haedi A. R. Zang S. S. (2024b). Efficacy of resveratrol supplementation on lipid profile parameters: an umbrella of meta-analysis. Prostagl. & Other Lipid Mediat. 175, 106903. 10.1016/j.prostaglandins.2024.106903 39255906

[B21] Liu Y. Han X. Bian Z. Peng Y. You Z. Wang Q. (2012). Activation of liver X receptors attenuates endotoxin-induced liver injury in mice with nonalcoholic fatty liver disease. Dig. Dis. Sci. 57, 390–398. 10.1007/s10620-011-1902-9 21948338

[B22] Madden A. M. Trivedi D. Smeeton N. C. Culkin A. (2017). Modified dietary fat intake for treatment of gallstone disease. Cochrane Database Syst. Rev. 10.1002/14651858.cd012608 PMC10845213 38318932

[B23] Mc Auley M. T. (2020). Effects of obesity on cholesterol metabolism and its implications for healthy ageing. Nutr. Res. Rev. 33, 121–133. 10.1017/S0954422419000258 31983354

[B24] Méndez-Sánchez N. Cárdenas-Vázquez R. Ponciano-Rodríguez G. Uribe M. (1996). Pathophysiology of cholesterol gallstone disease. Arch. Med. Res. 27, 433–441. 8987174

[B25] Méndez-Sánchez N. Zamora-Valdés D. Chávez-Tapia N. C. Uribe M. (2007). Role of diet in cholesterol gallstone formation. Clin. Chim. Acta 376, 1–8. 10.1016/j.cca.2006.08.036 17055469

[B26] Pan J. Zhu X. (2024). Etiology and treatment advances of hematochezia in infants aged ≤3 months. J. Biosci. Med. 12, 273–285. 10.4236/jbm.2024.1211023

[B27] Rozema E. Atanasov A. G. Fakhrudin N. Singhuber J. Namduang U. Heiss E. H. (2012). Selected extracts of Chinese herbal medicines: their effect on NF-κB, PPARα and PPARγ and the respective bioactive compounds. Evid. Based Complement. Altern. Med. 2012, 983023. 10.1155/2012/983023 PMC3366346 22675394

[B28] Shen S. Huang D. Qian S. Ye X. Zhuang Q. Wan X. (2023). Hyodeoxycholic acid attenuates cholesterol gallstone formation via modulation of bile acid metabolism and gut microbiota. Eur. J. Pharmacol. 955, 175891. 10.1016/j.ejphar.2023.175891 37429516

[B29] Silvain J. Nagaswami C. Weisel J. W. Collet J.-P. Montalescot G. Dhar G. (2012). The effect of ethanol on cholesterol crystals during tissue preparation for scanning electron microscopy: reply. J. Am. Coll. Cardiol. 59, 93. 10.1016/j.jacc.2011.08.065 22192678 PMC3711809

[B30] Stinton L. M. Myers R. P. Shaffer E. A. (2010). Epidemiology of gallstones. Gastroenterology Clin. N. Am. 39, 157–169. 10.1016/j.gtc.2010.02.003 20478480

[B31] Tang Q.-L. Lv Z. Yu Y. Wang B. Li H. Jin M. (2016). Mechanism of dahuang lingxian capsule for regulating and controlling expression of hepatocyte transporters and bile metabolism spectrum in gallstone mice. Zhongguo Zhong xi yi jie he za zhi Zhongguo Zhongxiyi Jiehe Zazhi= Chin. J. Integr. Traditional West. Med. 36, 953–959. 30640991

[B32] Unalp-Arida A. Ruhl C. E. (2024). Burden of gallstone disease in the United States population: prepandemic rates and trends. World J. Gastrointest. Surg. 16, 1130–1148. 10.4240/wjgs.v16.i4.1130 38690054 PMC11056655

[B33] Viennois E. Pommier A. J. Mouzat K. Oumeddour A. El Hajjaji F. Z. Dufour J. (2011). Targeting liver X receptors in human health: deadlock or promising trail? Expert Opin. Ther. Targets 15, 219–232. 10.1517/14728222.2011.547853 21204733

[B34] Vikal A. Maurya R. Bhowmik S. Khare S. Raikwar S. Patel P. (2024). Resveratrol: a comprehensive review of its multifaceted health benefits, mechanisms of action, and potential therapeutic applications in chronic disease. Pharmacol. Res. - Nat. Prod. 3, 100047. 10.1016/j.prenap.2024.100047

[B35] Wang H. Jiang C. Yang Y. Li J. Wang Y. Wang C. (2022). Resveratrol ameliorates iron overload induced liver fibrosis in mice by regulating iron homeostasis. PeerJ 10, e13592. 10.7717/peerj.13592 35698613 PMC9188311

[B36] Wang N. Tall A. R. (2003). Regulation and mechanisms of ATP-binding cassette transporter A1-mediated cellular cholesterol efflux. Arterioscler. Thromb. Vasc. Biol. 23, 1178–1184. 10.1161/01.ATV.0000075912.83860.26 12738681

[B37] Wang X. Yu W. Jiang G. Li H. Li S. Xie L. (2024). Global epidemiology of gallstones in the 21st century: a systematic review and meta-analysis. Clin. Gastroenterol. Hepatol. 22, 1586–1595. 10.1016/j.cgh.2024.01.051 38382725

[B38] Wen S.-H. Tang X. Tang T. Ye Z.-R. (2024). Association between weight-adjusted-waist index and gallstones: an analysis of the national health and nutrition examination survey. BMC Gastroenterol. 24, 40. 10.1186/s12876-024-03127-9 38238700 PMC10797852

[B39] Wen X. Zhang B. Wu B. Xiao H. Li Z. Li R. (2022). Signaling pathways in obesity: mechanisms and therapeutic interventions. Signal Transduct. Target Ther. 7, 298. 10.1038/s41392-022-01149-x 36031641 PMC9420733

[B40] Xia X. Jung D. Webb P. Zhang A. Zhang B. Li L. (2012). Liver X receptor β and peroxisome proliferator-activated receptor δ regulate cholesterol transport in murine cholangiocytes. Hepatology 56, 2288–2296. 10.1002/hep.25919 22729460 PMC3469731

[B41] Yang B. Cao P. Bao G. Wu M. Chen W. Wu S. (2024). Inhibiting miRNA-146a suppresses mouse gallstone formation by regulating LXR/megalin/cubilin-media cholesterol absorption. Heliyon 10, e36679. 10.1016/j.heliyon.2024.e36679 39296173 PMC11407981

[B42] Yue J. Li B. Jing Q. Guan Q. (2015). Salvianolic acid B accelerated ABCA1-dependent cholesterol efflux by targeting PPAR-γ and LXRα. Biochem. Biophys. Res. Commun. 462, 233–238. 10.1016/j.bbrc.2015.04.122 25956064

[B43] Zhu R. Ou Z. Ruan X. Gong J. (2012). Role of liver X receptors in cholesterol efflux and inflammatory signaling (review). Mol. Med. Rep. 5, 895–900. 10.3892/mmr.2012.758 22267249 PMC3493071

